# The Long and Winding Road to GUIDE Participation

**DOI:** 10.1002/alz.095811

**Published:** 2025-01-09

**Authors:** David B. Reuben

**Affiliations:** ^1^ University of California, Los Angeles, Los Angeles, CA USA

## Abstract

N/A N/A N/A N/A N/A N/A N/A N/A

